# Decreased survival of newborn neurons in the dorsal hippocampus after neonatal LPS exposure in mice^☆^

**DOI:** 10.1016/j.neuroscience.2013.08.040

**Published:** 2013-12-03

**Authors:** K. Järlestedt, A.S. Naylor, J. Dean, H. Hagberg, C. Mallard

**Affiliations:** a Department of Physiology, Institute of Neuroscience and Physiology, Sahlgrenska Academy, University of Gothenburg, Gothenburg, Sweden; b Centre for the Developing Brain, King’s College, Perinatal Imaging & Health, St Thomas’ Hospital, London, United Kingdom; c Perinatal Center, Department of Obstetrics and Gynecology, Institute of Clinical Sciences, Sahlgrenska Academy, University of Gothenburg, Gothenburg, Sweden

**Keywords:** BrdU, bromodeoxyuridine, CS, conditioned stimulus, DG, dentate gyrus, DCX, doublecortin, GCL, granule cell layer, LPS, lipopolysaccharide, NS, neutral stimulus, TFC, trace fear conditioning, TLR, toll-like receptor, US, unconditioned stimulus, LPS, neonatal, neurogenesis, stem cell

## Abstract

Experimental studies show that inflammation reduces the regenerative capacity in the adult brain. Less is known about how early postnatal inflammation affects neurogenesis, stem cell proliferation, cell survival and learning and memory in young adulthood. In this study we examined if an early-life inflammatory challenge alters cell proliferation and survival in distinct anatomical regions of the hippocampus and whether learning and memory were affected. Lipopolysaccharide (LPS, 1 mg/kg) was administered to mice on postnatal day (P) 9 and proliferation and survival of hippocampal cells born either prior to (24 h before LPS), or during the inflammatory insult (48 h after LPS) was evaluated. Long-term cell survival of neurons and astrocytes was determined on P 41 and P 60 in the dorsal and ventral horns of the hippocampus. On day 50 the mice were tested in the trace fear conditioning (TFC) paradigm. There was no effect on the survival of neurons and astrocytes that were born before LPS injection. In contrast, the number of neurons and astrocytes that were born after LPS injection were reduced on P 41. The LPS-induced reduction in cell numbers was specific for the dorsal hippocampus. Neither early (48 h after LPS) or late (33 days after LPS) proliferation of cells was affected by neonatal inflammation and neonatal LPS did not alter the behavior of young adult mice in the TFC test. These data highlight that neonatal inflammation specifically affects survival of dividing neurons and astrocytes, but not post-mitotic cells. The reduction in cell survival could be attributed to less cell survival in the dorsal hippocampus, but had no effect on learning and memory in the young adult.

## Highlights

• Neonatal inflammation reduces the survival of dividing neurons and astrocytes.
• Neonatal inflammation does not affect the survival of post-mitotic cells.
• Decrease in cell survival was specific for the granule cells of the dorsal blade of the hippocampus.

## Introduction

The neonatal period in humans and rodents is a fundamental phase in normal brain development. Neurogenesis, synaptogenesis and neuronal connectivity are prominent features of the developing neonatal hippocampus. The hippocampus plays a key role in learning and memory and the birth of new granule neurons in the dentate gyrus (DG) of the hippocampus in both rodents and humans is assumed to be important for maintaining memory function throughout life (Kuhn et al., 1996; Eriksson et al., 1998). Thus, disturbance of neurogenesis during a sensitive period of brain development may be one of the causes of long-lasting effects on behavioral and cognitive functions (Hornig et al., 1999; Shi et al., 2003).

Inflammation in the adult brain is known to impair neurogenesis and decrease the number of immature neurons in the hippocampus (Ekdahl et al., 2003; Monje et al., 2003). More specifically, it was shown that lipopolysaccharide (LPS), a component of Gram-negative bacteria, did not affect proliferation of cells but reduced the survival of newly generated neurons in the adult hippocampus (Bastos et al., 2008). In the developing neonatal brain, systemically administered LPS induces a strong inflammatory response (Eklind et al., 2006). Prenatal LPS-induced inflammation decreases neurogenesis in mice pups up to 14 days after birth (Cui et al., 2009) and LPS administration to early postnatal mice provokes hippocampal seizures (Auvin et al., 2007; Galic et al., 2008). Less is known about the effect of early postnatal LPS exposure on neurogenesis, stem cell proliferation, survival and learning and memory in young adulthood.

Here we investigated the effect of cell proliferation, survival and differentiation before and after an inflammatory insult. We injected mice with LPS on postnatal day (P) 9 and injected bromodeoxyuridine (BrdU) either at P8 (24 h prior to the inflammatory insult) or at P11 (48 h after the inflammatory insult) and investigated cell survival in the dorsal and ventral hippocampi. In addition, an associative learning task was used to evaluate the behavioral effect of neonatal LPS.

## Experimental procedures

### Animals

Male/female C57/BL6 mice pups (Charles River Breeding Laboratories, Germany) were used for all experiments. All animals were housed at Experimental Biomedicine, University of Gothenburg at constant temperature (24 °C) with a relative humidity of 50–60%. A 12-h dark/light cycle was maintained with lights on from 19:00 to 07:00 with food and water available *ad libitum*. All procedures described were conducted in accordance with and approved by the Ethics Committee for the University of Gothenburg (ethical number 277/07 and 374/09).

### Methods

#### Effect of neonatal LPS on progenitor proliferations and cell survival in hippocampus in the young adult

**Fig. 1:**
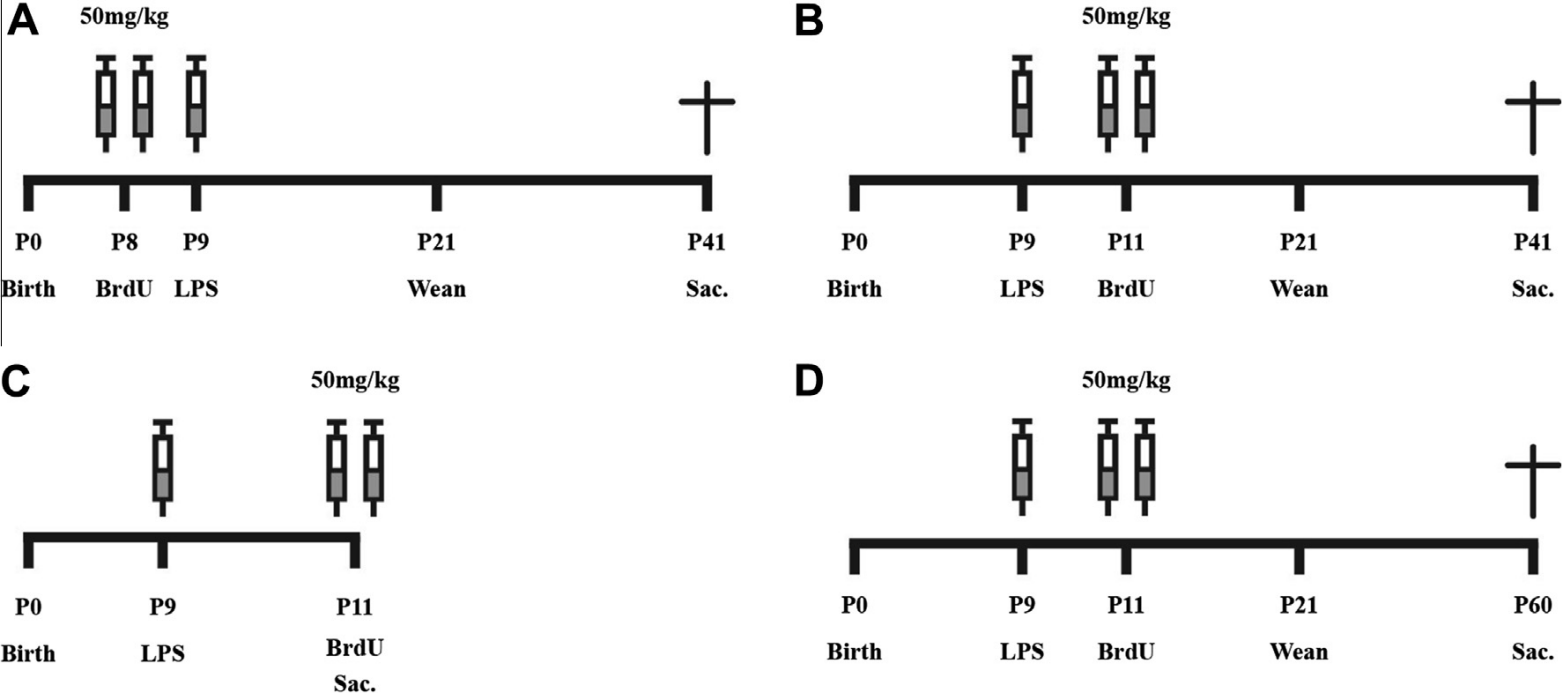
Schematic diagram of the experimental procedures. (A) To investigate the effect of hippocampal cell survival on cells undergoing division prior to an inflammatory response, mice (*n* = 10 per group) were injected with BrdU (50 mg/kg) at P8 and then injected with saline or LPS (1 mg/kg). Thirty-three days later, animals were perfused and brains removed for immunohistochemistry. (B) To investigate the effect of hippocampal cell survival on cells undergoing division during an inflammatory response, mice (*n* = 10 per group) were injected with saline or LPS (1 mg/kg) at P9 and then injected with BrdU (50 mg/kg) at P11. Thirty days later animals were perfused and brains removed for immunohistochemistry. (C) To investigate the effects of LPS on hippocampal progenitor proliferation, mice (*n* = 10 per group) were injected with saline or LPS (1 mg/kg) at P9 and then injected with BrdU (50 mg/kg) at P11. Two hours after injection of BrdU the animals were perfused and brains removed for immunohistochemistry. (D) To investigate the effect of hippocampal cell survival in the dorsal respectively ventral horn, mice were injected with saline (*n* = 10) or LPS (1 mg/kg, *n* = 13) at P9 and then injected with BrdU (50 mg/kg) at P11. Forty-nine days later animals were perfused and brains removed for immunohistochemistry.

To investigate the effect of LPS on hippocampal cell survival in the young postnatal mouse, pups (*n* = 10 in each group) were injected with a single dose of LPS (1 mg/kg, ultra pure *Escherichia coli* LPS 055:B5, Sigma–Aldrich Sweden AB Stockholm, Sweden) or saline i.p. at postnatal day (P) 9. Cells undergoing division were labeled at either P8 (24 h prior to LPS injection), to investigate the effect of LPS on cells undergoing division prior to inflammation or at P11 (48 h post LPS injection) to investigate cells undergoing cell division post injection of LPS. BrdU (50 mg/kg/injection, Sigma–Aldrich) was injected in two pulses during the first few hours of the dark phase and during the middle of the dark phase (10 am and 2 pm, respectively). Animals were then left in their home cages and were sacrificed 30 days post BrdU injection (Fig. 1A, B).

To investigate the effect of LPS on progenitor proliferation during an ongoing inflammatory period, separate mice were injected with a single dose of LPS (1 mg/kg, *n* = 10) or saline (*n* = 10), i.p. at P9 and then with BrdU (50 mg/kg) at P11 before being sacrificed 2 h after the last BrdU injection (Fig. 1C).

#### Effect of neonatal LPS on regional cell survival in the hippocampus of adults

It is believed that the dorsal and ventral poles of the hippocampus serve different functions, where particularly lesions in the dorsal horn impair retention of contextual memory (Kim and Fanselow, 1992). In order to investigate whether inflammation has regional effects on hippocampal cell survival we injected P9 mice with saline (*n* = 10) or LPS (*n* = 13) at P9 and BrdU at P11. Animals were sacrificed at P60 and the dorsal and ventral hippocampi analyzed separately as described below.

#### Effect of neonatal LPS on learning and memory in early adulthood

To investigate the effect of neonatal LPS on learning and memory in early adulthood we tested the mice in the trace fear conditioning (TFC) paradigm as previously described (Jarlestedt et al., 2011). TFC involves pairing a neutral stimulus (NS) with an aversive stimulus (unconditioned stimulus, US). The NS consisted of a 20-s tone (80 dB, 670 Hz) and the US of a 2-s foot shock (0.5 mA). Once the animal has learned the association between the NS and the US, the NS becomes a conditioned stimulus (CS) and the CS alone will elicit a conditioned response such as freezing behavior. Freezing was defined as a complete lack of movement and was scored visually by the experimenter, who was blinded to experimental group identity. Pups were injected with a single dose of LPS (1 mg/kg, *n* = 10) or saline (*n* = 10) i.p. at P9 and their performance in TFC was tested in an automatic reflex conditioner chamber (UgoBasile, No. 7530). On the first day, at P49, the animals were first scored for baseline freezing in the conditioning box for 2 min, and then the shock-paired tone (80 dB, 670 Hz) was turned on for 20 s, followed by a 2-s delay and then a 2-s shock (0.5 mA). The animals were kept in the conditioning box for 30 s to allow for consolidation of information about the shock-paired context after the shock. The day after conditioning, at P50, freezing behavior was measured for 2 min in the conditioning box. The shock-paired tone was then presented for 30 s, and freezing behavior was measured for 2 min following the tone presentation Freezing behavior was quantified by scoring freezing as present or absent once every 10 s, and percentage freezing over a 2-min period was calculated.

### Immunohistochemistry

Animals were deeply anesthetized and intracardially perfused with saline and 5% buffered formaldehyde (Histofix; Histolab, Sweden). Brains were rapidly removed and immersion fixed in 5% formaldehyde for 24 h. Consecutive 25-μm-thick free-floating sections were used in all experiments. In the experiments on cell survival and proliferation at P11 and P41, sections were cut in the sagittal plane and in the study to examine cell survival in dorsal and ventral hippocampi specifically; sections were cut in the coronal plane.

For BrdU, DNA dentaturation was conducted by incubation for 30 min in 2 N hydrochloric acid at 37 °C, followed by 10 min in 0.1 N borate buffer (pH 8.5). After washing, sections were incubated for 30 min in 0.6% H_2_O_2_, blocked with 3% normal donkey serum in 0.1% Triton X-100, then incubated with monoclonal anti-BrdU (1:500; Nordic Biosite, Sweden) overnight at 4 °C. Sections were then washed in TBS, placed in the secondary antibody (biotinylated donkey anti-mouse; 1:1000; Jackson ImmunoResearch Laboratories, West Grove, PA, USA) followed by amplification with avidin–biotin complex (Vectastain ABC Elite, Vector laboratories) and then visualized using a detection solution (0.25 mg/ml diaminobenzidine, Saveen Biotech AB, Sweden). Doublecortin (DCX) and Anti-phospho-Histone H3 were pretreated with sodium citrate (pH 9) for antigen retrieval and then sections were washed, incubated for 30 min in 0.6% H_2_O_2_, blocked with 3% normal donkey serum in 0.1% Triton X-100, then incubated in polyclonal goat-anti DCX (1:250; Santa Cruz, CA) or polyclonal phospho-Histone H3 (1:1000; Upstate Bio) overnight at 4 °C. Sections were then washed in TBS, placed in the secondary antibody (biotinylated donkey anti-goat or biotinylated donkey anti-rabbit; 1:1000; Jackson ImmunoResearch Laboratories, West Grove, PA) followed by amplification with avidin–biotin complex (Vectastain ABC Elite, Vector laboratories) and then visualized using a detection solution (0.25 mg/ml diaminobenzidine, Saveen Biotech AB, Malmö, Sweden). For double-immunolabeling, free-floating sections were incubated in a mixture of primary antibodies, anti-BrdU (1:250) and IBA-1 (1:500) raised in different species for 72 h at 4 °C. Sections were then washed and visualized using appropriate Alexa Fluor conjugated secondary antibodies (1:1000; Molecular Probes, Eugene, OR, USA). Sections were mounted on slides in fluorescent medium containing DAPI (DAPI Pro-Long Gold anti-fade reagent, Molecular Probes, Eugene, OR, USA).

### Stereological quantification of cells

Every 10th section, for animals sacrificed at P11 and every 12th section, for animals sacrificed at P41 and P60 throughout the hippocampus was used to determine the total number of BrdU and DCX-labeled cells in the DG (subgranular zone (SGZ) and granule cell layer (GCL)) under light microscopy in each animal. The percentage number of newborn neurons (60 BrdU-positive cells per animal) was assessed using a confocal microscope (Leica TCS SP2, Leica Microsystems, Germany). The resulting percentages of NeuN-positive cells were multiplied with the absolute number of BrdU-positive cells to give the absolute number of newly generated neurons. The volume of the GCL was measured in every 12th section throughout the hippocampus and the total sum of the area traced was multiplied by section thickness and series number to give the volume of the entire GCL.

#### Statistical analysis

Values are expressed as mean ± standard error of the mean (SEM). Data were analyzed using an independent-*T*-test to determine differences between groups.

## Results

### Normal survival of cells in the granule cell layer that were born prior to injection of LPS

**Fig. 2:**
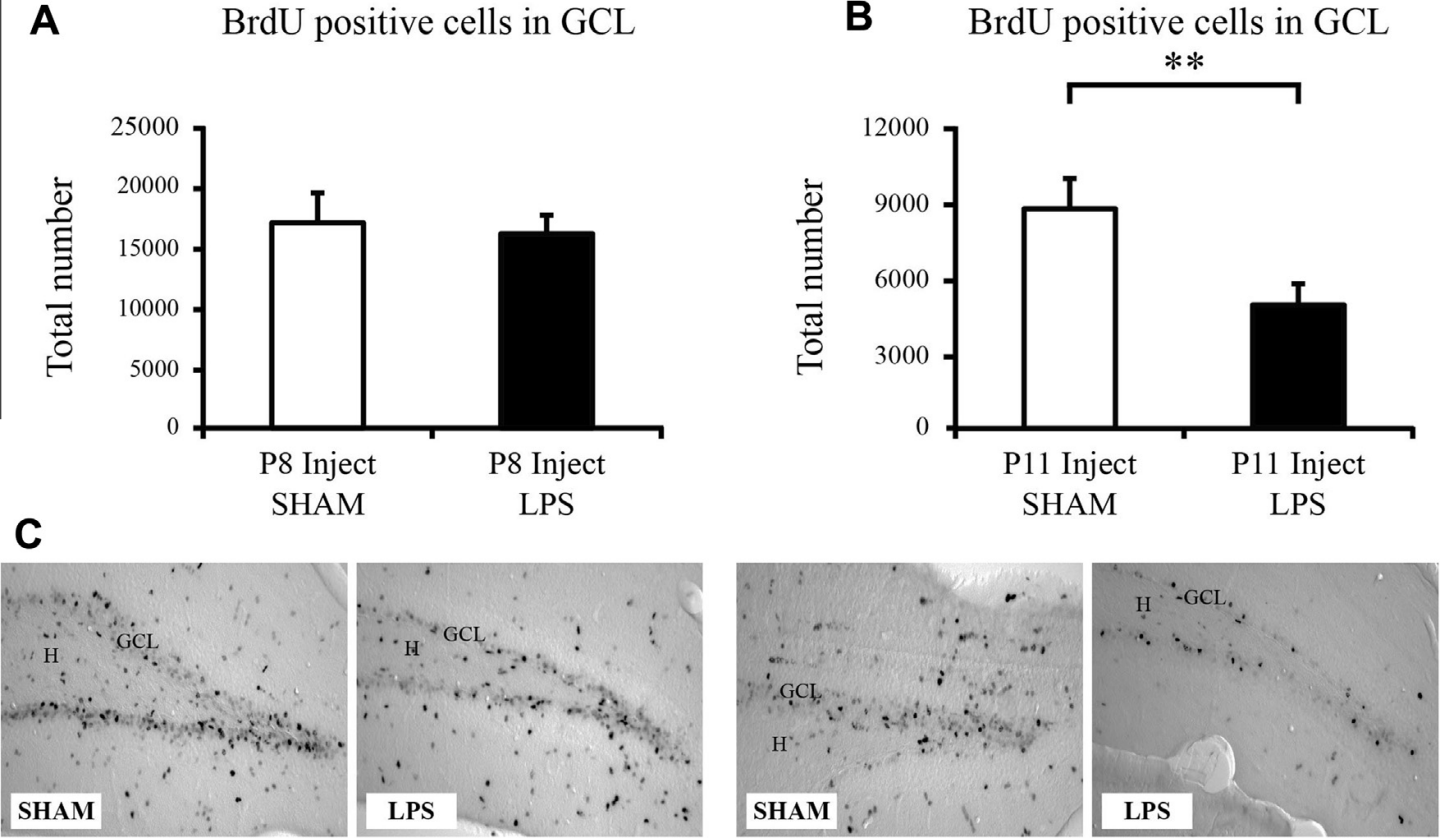
Progenitor cell differentiation in the mouse dentate gyrus. (A, C) Injection of BrdU 24 h prior to LPS, in order to investigate the effect of cells born prior to an inflammatory response, revealed no difference in the total number of cells that survived 30 days post injection. (B, D) Injection of BrdU 48 h after injection of LPS revealed a significant decrease on the survival of cells that were born during the inflammatory period. Data are represented as group means ± SEM. ^∗∗^*P* < 0.01. Hilus (H); Granule cell layer (GCL).

Mice (*n* = 10 per group) were injected with BrdU at P8 and 24 h later injected with LPS or saline at P9. Animals were then left for 33 days post BrdU injection to analyze the effect of LPS on the survival of cells born 24 h before an inflammatory insult (Fig. 1A). There was no significant difference between saline- and LPS-injected animals in the number of cells that survived 33 days post BrdU injection in the GCL of the hippocampus (saline: 16,982 ± 2481 vs. LPS: 16,086 ± 1530; *p* > 0.05, Fig. 2A, B). We also found no effect on the volume of the GCL after administration of LPS (saline: 0.064 ± 0.004 mm^3^ vs. LPS: 0.054 ± 0.005 mm^3^, *p* > 0.05).

**Fig. 3:**
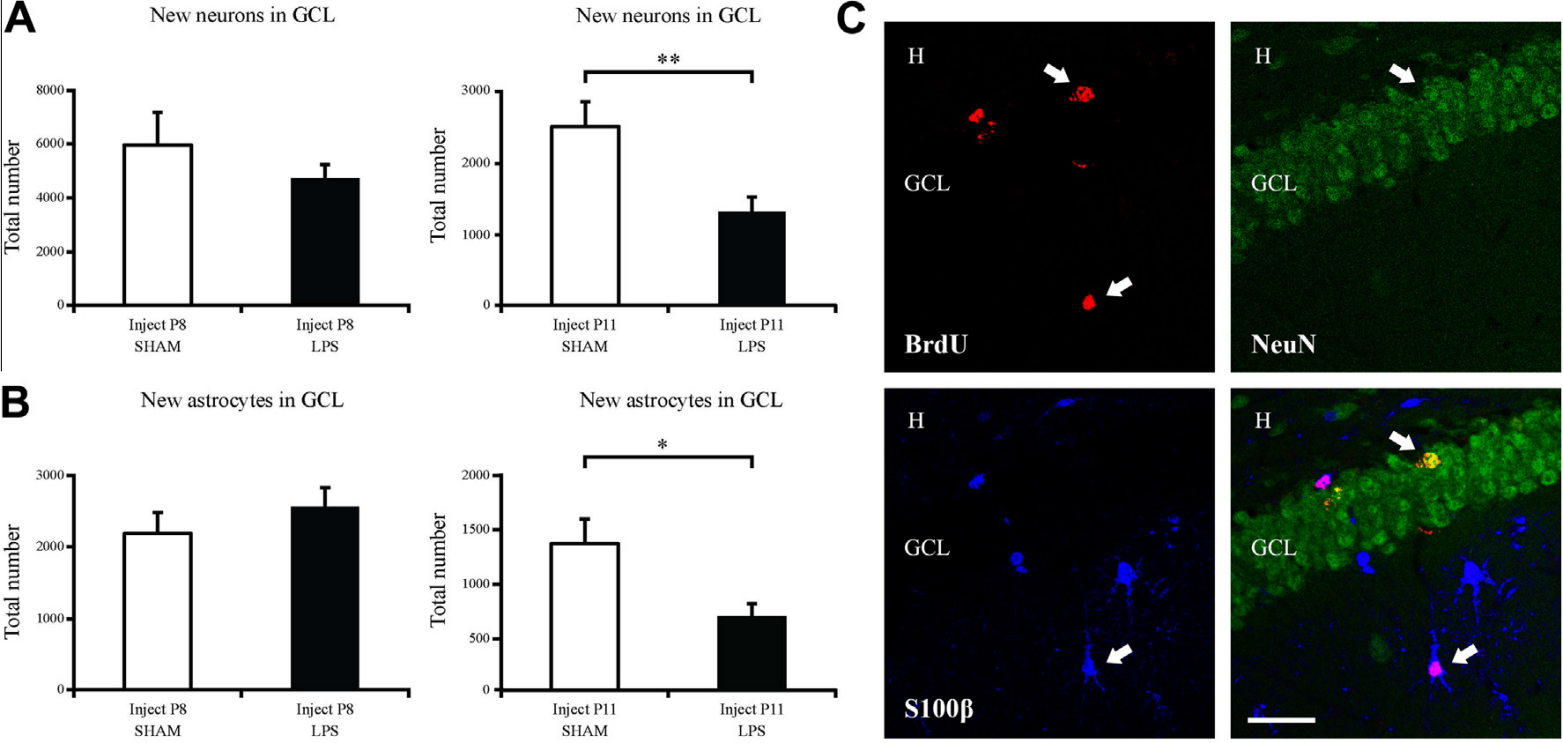
LPS decreases the number of newborn neurons and astrocytes on cells born during the inflammatory response. (A, C) Confocal microscope analysis (BrdU-positive cells (red), NeuN-positive cells (green)) revealed no changes in the number of newborn neurons in cells born prior to an inflammatory response. However, if cells were born during a period of inflammation, there was a significant decrease in the number of newborn neurons. (B, C) Confocal microscope analysis (BrdU-positive cells (red), S100β-positive cells (blue)) revealed no changes in the number of newborn astrocytes in cells born prior to an inflammatory response. However, if cells were born during a period of inflammation, there was a significant decrease in the number of newborn astrocytes. Group means ± SEM. Scale bar = 50 μm. ^∗^*P* < 0.05, ^∗∗^*P* < 0.01. Arrows pointing to the right indicate BrdU-positive neuron and arrows pointing to the left indicate BrdU-positive astrocyte. (For interpretation of the references to color in this figure legend, the reader is referred to the web version of this article.)

Neurogenesis was assessed by the analysis of BrdU in combination with the neuronal marker NeuN. There was no significant difference in the percentage of new cells that became neurons in the saline compared to LPS-treated groups (data not shown). There was also no difference in the total number of newborn neurons in the GCL (saline: 5686 ± 1158 vs. LPS: 4482 ± 518; *p* > 0.05, Fig. 3A, C). The number of newborn astrocytes were also analyzed using BrdU and the astrocyte marker S100β. There was no significant difference in the total number of newborn astrocytes in the GCL (saline: 858 ± 286 vs. LPS: 854 ± 270; *p* > 0.05, Fig. 3B, C).

### Reduced survival of cells in the granule cell layer that were born after injection of LPS

Mice (*n* = 10 per group) were injected with BrdU at P11, 48-h post injection of LPS or saline at P9. Animals were then left for 30 days post BrdU injection to analyze the effect of LPS on the survival of cells born after an inflammatory insult (Fig. 1B). There was a significant decrease in the total number of BrdU-positive cells that survived after injection of LPS (saline: 8714 ± 1 202 vs. LPS: 4899 ± 776; *P* < 0.05; Fig. 2B, C). We found no effect on the volume of the GCL after the administration of LPS (saline: 0.060 ± 0.003 mm^3^ vs. LPS: 0.057 ± 0.004 mm^3^, *p* > 0.05).

Neurogenesis was assessed by the analysis of BrdU in combination with the neuronal marker NeuN. There was no significant difference in the percentage of new cells that became neurons after LPS (data not shown). However, there was a decrease in the total number of new neurons born in the GCL after LPS (saline: 2446 ± 344 vs. LPS: 1285 ± 197; *P* < 0.01; Fig. 3A, C). Analysis of the total number of newborn astrocytes also revealed a decrease after injection of LPS (saline: 1504 ± 253 vs. LPS: 754 ± 134; *P* < 0.05; Fig. 3B, C).

### No effect of LPS on progenitor proliferation in the granule cell layer 48 h after injection of LPS

**Fig. 4:**
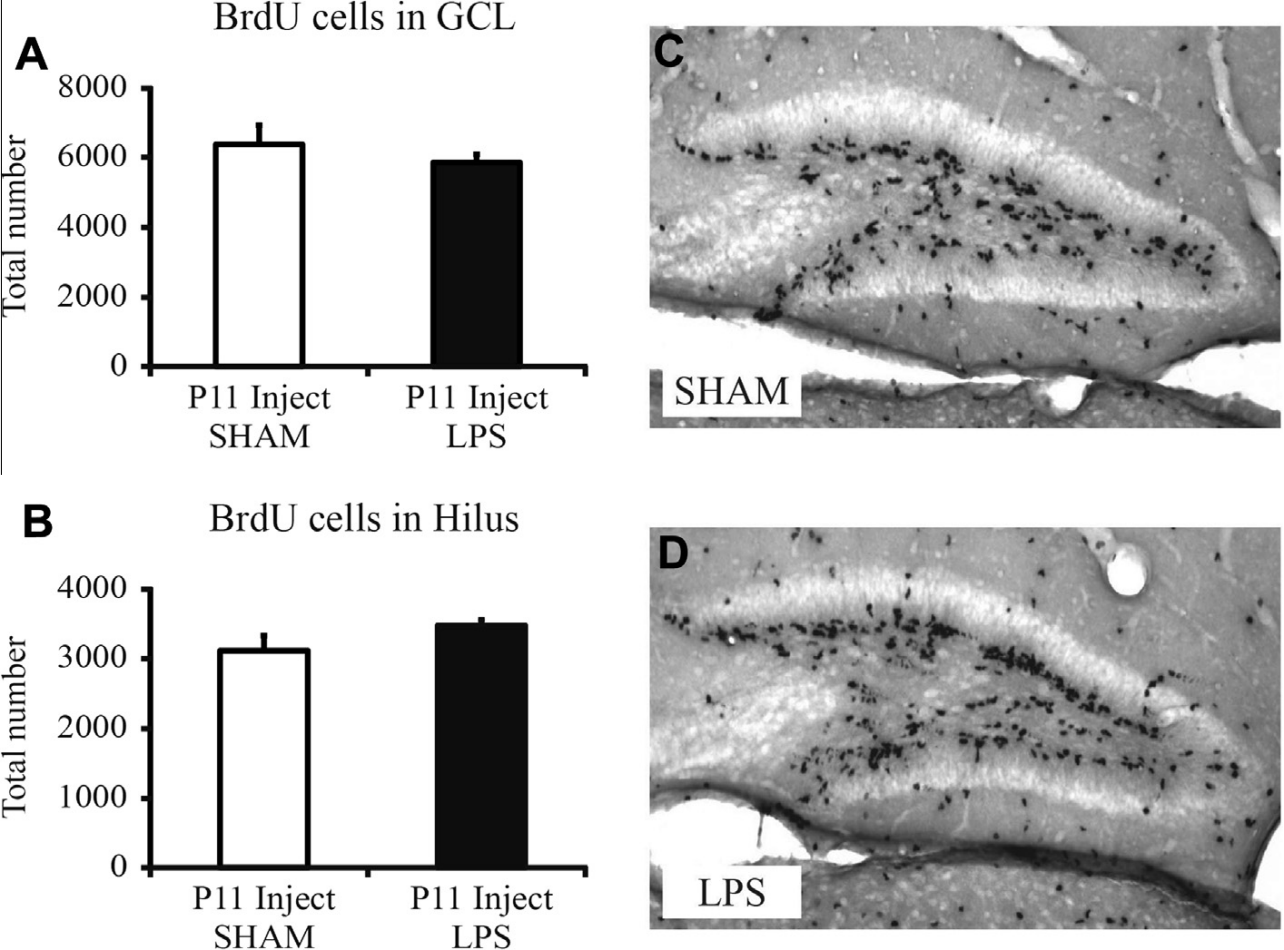
Assessment of cell proliferation in the dentate gyrus 48 h after injection of LPS. (A, C, D) Proliferating cells in the granule cell layer, labeled with BrdU at P11, 48 h post injection of LPS, were not decreased after LPS injection compared to saline-injected animals. (B–D) Proliferating cells in the Hilus were not decreased after LPS injection compared to saline-injected animals. Data are represented as group means ± SEM. Scale bars = 100 μm.

To further understand the effect of cell survival on cells born after LPS, we analyzed the effect of LPS administration on cell division and proliferation. Mice (*n* = 10 per group) were injected with LPS or saline at P9 and BrdU was administered at P11, with the animals sacrificed 2 h after the last BrdU injection (Fig. 1C). We found no effect of LPS on the total number of newborn proliferating cells in the GCL (saline: 6237 ± 541 vs. LPS: 5730 ± 233; *p* > 0.05, Fig. 4A, B) or in the hilus (saline: 3047 ± 210 vs. LPS: 3402 ± 77; *p* > 0.05, Fig. 4C, D).

### No effect on total numbers of newborn and resident microglia in the granule cell layer 48h after injection of LPS

To further understand the immediate effects of LPS on the hippocampal microenvironment, we analyzed the total number of resident microglia in the GCL and the total number of newborn resident microglia in the GCL using the microglial marker IBA-1 and BrdU 48 h after LPS (Fig 1C). We found no significant effect on the total number of IBA-1-positive resident microglia after LPS in the GCL (saline: 737 ± 94 vs. LPS: 915 ± 65, *p* > 0.05). There were also very few newborn BrdU/IBA-1-positive cells in the GCL 48 h after LPS with no significant difference compared to saline-administered animals (data not shown).

### No long-term effect of LPS on immature neurons or proliferation in the adult granule cell layer

We evaluated the effects of LPS on proliferation in the adult GCL (1 month post BrdU injection) using the proliferative marker phospho-histone H3. We found no significant difference in the number of proliferative cells in the GCL in animals administered with LPS compared to saline controls (saline: 1956 ± 145 vs. LPS: 1915 ± 199, *p* > 0.05). We also analyzed the number of immature neurons using DCX. We found no effect of LPS on the number of immature neurons at the time of sacrifice (saline: 14,649 ± 637 vs. LPS: 14,097 ± 892, *p* > 0.05).

### Neonatal LPS selectively reduces cell survival in the dorsal granule cell layer at P60

**Fig. 5:**
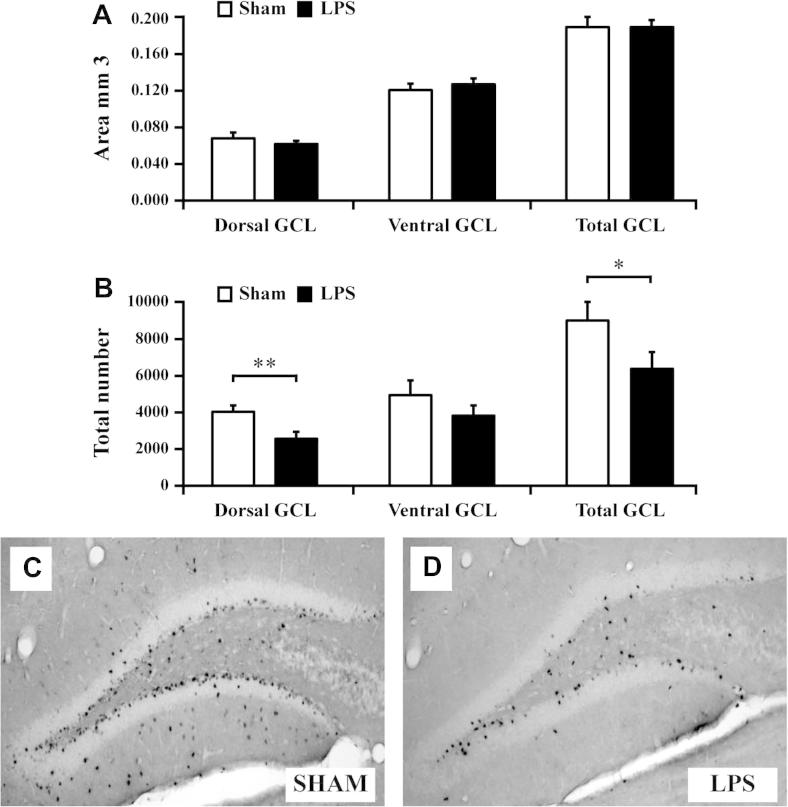
LPS reduces the survival of newborn cells specifically in the dorsal hippocampus. (A) The area of the dorsal and ventral granule cell layer (GCL) was similar in LPS-exposed pups compared to saline control at P60. (B–D) Survival of BrdU-positive cells, labeled at P11, 48 h after LPS, was significantly reduced in the dorsal hippocampus at P60 compared to sham animals. (B) There was no effect by neonatal LPS on survival of BrdU labeled cells in the ventral hippocampus at P60. Group means ± SEM. ^∗^*P* < 0.05, ^∗∗^*P* < 0.01.

Lesions in the dorsal hippocampus have been associated with learning deficits. We therefore investigated cell survival in dorsal and ventral hippocampi respectively at P60 following neonatal (P9) LPS (*n* = 13) or saline (*n* = 10) administration (Fig 1D). There was no difference in the area of either the dorsal or ventral GCL in LPS-exposed pups compared to saline control at P60 (Fig 5A, *p* > 0.05). In contrast, the survival of BrdU-positive cells was significantly reduced in the dorsal hippocampus at P60 following neonatal LPS (saline: 4029 ± 357, *n* = 10 vs. LPS 2570 ± 374, *n* = 13, *P* < 0.01, Fig 5B, C, D), but not in the ventral hippocampus (Fig 5B, *p* > 0.05).

### No effect of neonatal LPS on learning and memory at P60

To investigate the animals’ learning and memory ability in young adulthood following neonatal LPS, we tested the animals’ performance in the TFC at P60. Neither freezing behavior in response to the conditioning box, as a measure of contextual memory, (saline: 48.81 ± 10.51 vs. LPS: 55.21 ± 10.90, *p* > 0.05) nor freezing in response to the CS, i.e. tone (saline: 58.33 ± 10.56 vs. LPS 60.42 ± 11.44, *p* > 0.05) was affected by neonatal LPS.

## Discussion

The present study demonstrates that there are distinct differences in the survival of cells dividing before and after an inflammatory insult. While the survival of cells born before injection of LPS was not altered, cells that were born 48 h after injection of LPS had a decreased survival rate compared to control animals. However, levels of proliferation were normal after the inflammatory insult, indicating that LPS has stronger effects on cell survival than on proliferation during inflammation in the neonatal mouse. The reduction in cell survival could be attributed to less cell survival specifically in the dorsal hippocampus, but had no effect on learning and memory.

Systemic LPS is a strong inducer of inflammation in prenatal, immature, juvenile and adult models of infection, resulting in a myriad of inflammatory cytokines and effects on immune cells in the brain (Cai et al., 2000; Eklind et al., 2006; Qin et al., 2007; Wang et al., 2009; Dean et al., 2010). Neurogenesis is particularly susceptible to inflammatory processes, evidenced by the effects seen in adult and prenatal studies of infection/inflammation on decreases in cell proliferation and neurogenesis (Ekdahl et al., 2003; Monje et al., 2003; Cui et al., 2009). We found a strong effect on cell survival of cells that were born after injection of LPS (LPS at P9, BrdU at P11). However, there was no effect on cell survival in cells that were born before LPS injection (BrdU injected at P8, LPS at P9). These results indicate that newborn cells having undergone cell division are afforded a protection that does not alter cell survival, while cells that are born into an inflamed environment are more vulnerable.

Interestingly, we found no effect on global cell proliferation during the inflammatory period (P11), which indicates that LPS has a greater effect on the survival of cells in the hippocampus than on cell proliferation. Further, we found no long-term effect on numbers of immature neurons or proliferation 30 days after LPS administration (P41). This indicates that LPS administration at P9 has an almost exclusive effect on cell survival but no effect on cell proliferation after 48 h or up to 30 days post inflammation exposure. This is in line with data from studies in the adult brain where LPS induced an increased cell death in the DG, but had no effect on proliferation, suggesting that LPS mainly impaired cell survival (Bastos et al., 2008). However, as our study does not provide direct evidence of increased cell death, there is a possibility that the reduced number of cells could also be due to that the progenitors of the new neurons underwent reduced number of amplifying divisions prior to differentiation.

The underlying mechanisms of LPS-induced reduction in cell survival are unknown. The LPS receptor, toll-like receptor (TLR)-4, is known to affect neurogenesis in the adult (Rolls et al., 2007). Cell culture experiments demonstrate that LPS-stimulated microglia increases neuronal and oligodendoglia cell death, which is mediated through the TLR-4 receptor and the TLR-4 adaptor protein MyD88 (Lehnardt et al., 2003; Dean et al., 2010). Combined these studies indicate a potential role for TLR-4 mediated pathways in the reduced cell survival in the present investigation.

Glia cells are important regulators of neurogenesis. In the adult brain, hippocampal astrocytes actively regulate neurogenesis both by promoting proliferation and differentiation of neural stem cells (Song et al., 2002). *In vitro* experiments have shown that media from LPS-stimulated astrocytes increases neural progenitor cell proliferation but decreases neurogenesis (Go et al., 2009). Further, media from LPS-stimulated microglia increases oxidative stress-induced (H_2_O_2_) death in astrocyte-rich cultures (Correa et al., 2011) and studies *in vivo* in sheep demonstrate reduced number of glia cells and neurons following LPS exposure in utero (Dean et al., 2011). Thus these studies support the reduced survival of both astrocytes and neurons following LPS injection at P11 that we observed in the present study.

We did not see an increase in either the total number of newborn or resident microglia in the granule cell layer 2 days after LPS. In fact, there were very few Iba-1/BrdU-positive cells, which could be due to that the 2-h time point (after BrdU injection) was too early to investigate microglia proliferation. However, we have previously shown that peripheral LPS injection during the perinatal period induces a strong upregulation of inflammatory genes in the brain already 2 h after LPS (Eklind et al., 2006). More, specifically, we have recently observed both short-term (48 h) and long-term effects on microglia proliferation and activation in the hilus after LPS injection at P5, but with very few microglia cells present in the granule cell layer (Smith et al., unpublished observation). In support, a recent study shows that *E. coli* injection at P4 induces an increase in resident microglia and proliferating microglia in the CA1 and CA3 regions of the hippocampus, but not in the DG (Bland et al., 2010). Thus it seems that there are regional differences in microglia proliferation after LPS where inflammatory changes are more prominent in other regions of the hippocampus than specifically in the granule cell layer. Speculatively, microglia-mediated effects on neuronal cell proliferation in this brain region may therefore be indirect via factors released into the granule cell layer environment.

The dorsal and ventral hippocampi have been suggested to be functionally distinct structures (Moser and Moser, 1998; Fanselow and Dong, 2010). Spatial and contextual memory is associated with the dorsal hippocampus (Moser et al., 1995), while ventral horn lesions alter stress responses (Henke, 1990). In order to investigate the effects of inflammation on these distinct structures we assessed cell survival in dorsal and ventral hippocampi at P60 following neonatal LPS. We found that cell survival was only impaired in the dorsal horn, while there was no difference in the number of surviving cells in the ventral granule cell layer. Dorsal lesions have been associated with impairment in retention of contextual as opposed to cued fear (Kim and Fanselow, 1992). Thus our regional cell survival data prompted us to investigate potential functional consequences associated with dorsal hippocampal impairment using the fear conditioning test that offers both a test of spatial (context fear) and nonspatial (cued fear) memory where performance is motivated by emotion. However, in this study reduced cell survival in the dorsal hippocampus did not affect either contextual or cued fear memory. It is possible that more distinct lesions in the granule cell layer are required to elicit memory dysfuntion or others have shown that it is particularly injury to CA1 and CA3 that is associated with memory loss (Hunsaker and Kesner, 2008). Furthermore, a caveat of the study is that memory performance was assessed only at one time point. Potentially, there may be earlier transient effects that were not detected by the current protocol.

## Conclusion

We show a novel approach to understanding the effect of LPS on the survival of cells born in the hippocampus. By labeling newborn cells prior to inflammation we were able to show that survival of cells that have undergone division was not affected by injection of LPS, although the study did not address whether structural or functional integration may have been altered. However, survival of cells that undergo division during the inflammatory cascade that accompanies the administration of LPS was decreased. These detrimental effects of LPS were localized to the dorsal, but not ventral hippocampus. Although, we did not find that reduced cell survival resulted in deficits in learning and memory as tested in the TFC paradigm, the studies suggest that the developing brain, where granule cell proliferation is high, may be particularly sensitive to inflammation.
